# Oyster (*Crassostrea gigas*) Extract Attenuates Dextran Sulfate Sodium-Induced Acute Experimental Colitis by Improving Gut Microbiota and Short-Chain Fatty Acids Compositions in Mice

**DOI:** 10.3390/foods11030373

**Published:** 2022-01-27

**Authors:** Tatsuya Ishida, Hiroyuki Matsui, Yoshikazu Matsuda, Ryota Hosomi, Takaki Shimono, Seiji Kanda, Toshimasa Nishiyama, Kenji Fukunaga, Munehiro Yoshida

**Affiliations:** 1 Central Research Institute, Japan Clinic Co., Ltd., 1 Nishimachi, Taishogun, Kyoto 603-8331, Japan; tatsuya.ishida@japanclinic.co.jp (T.I.); hiroyuki.matsui@japanclinic.co.jp (H.M.); yoshikazu.matsuda@japanclinic.co.jp (Y.M.); 2 Faculty of Chemistry, Materials, and Bioengineering, Kansai University, 3-3-35 Yamate-cho, Osaka 564-8680, Japan; fukunagk@kansai-u.ac.jp (K.F.); hanmyou4@kansai-u.ac.jp (M.Y.); 3 Department of Hygiene and Public Health, Kansai Medical University, 2-5-1 Shin-machi, Osaka 573-1010, Japan; shimonot@hirakata.kmu.ac.jp (T.S.); kandas@hirakata.kmu.ac.jp (S.K.); tnishi@takii.kmu.ac.jp (T.N.)

**Keywords:** oyster extract, *Crassostrea gigas*, inflammatory bowel disease, experimental colitis, microbiota, short-chain fatty acid

## Abstract

Drugs for inflammatory bowel diseases can be associated with serious side effects, and the development of alternative candidate resources derived from natural products has attracted considerable attention. Oyster extract (OE) derived from *Crassostrea gigas* contains glycogen, taurine, and amino acids, and has been assigned diverse health-promoting properties. This study investigated the anti-colitis effect of OE intake on fecal microbiota and its metabolites of acute experimental colitis mouse model induced by dextran sulfate sodium (DSS). C57BL/6J mice (male) were divided into three groups: (1) American Institute of Nutrition (AIN) 93G diet + DSS-untreated, (2) AIN93G diet + DSS-treated, and (3) 5% OE diet + DSS-treated. Mice were fed each diet for 21 days, and then administered 2.5% DSS solution to induce acute colitis for 7 days. In DSS-induced colitis mice, OE decreased body weight loss and increased disease activity index during the DSS-induced period. In addition, OE tended to decrease the colon length shortening and the relative spleen weight and alleviated colonic tissue damage. Moreover, OE improved fecal short-chain fatty acids compositions and altered the structure of fecal microbiota. These results provide insight into the health-promoting property of OE in alleviating DSS-induced acute colitis, providing a basis for the development and use of functional foods.

## 1. Introduction

Inflammatory bowel diseases (IBD) are chronic inflammatory disorders characterized by chronic epithelial damage and relapsing/remitting inflammation of the colon [1]. IBD is a critical health problem around the world [2,3]. The pathogenesis of IBD is multifactorial, including alterations microbiota and its metabolites in the gut, defects in the epithelial barrier, immune dysregulation, environmental factors, and genetic susceptibility [4]. Anti-inflammatory drugs, especially steroids and anti-cytokines, are known to be effective in patients with IBD. However, with time, drug-induced remission weakens and is accompanied by side effects [5]. Hence, natural products have been attracting attention as alternatives to drugs and are being explored as alternative treatments to enhance conventional IBD therapy [6]. These alternative therapies are of great interest because of their low side effects [7]. Among these, algal oil [8], propolis extract [9], peptides derived from tuna [10], taurine [11], and astragalin [12] have been reported to exert protective effects against experimental colitis induced by dextran sulfate sodium (DSS) in mice. Thus, the potential exists for IBD patients to have a greater variety of substances that are effective, in the form of alternative treatments.

Oysters (*Crassostrea gigas*) contain a variety of nutrients and oyster extract (OE) is prepared by extracting raw oysters with hot water and then drying them. The main components of OE include glycogen, taurine, zinc, protein, and amino acids. Previous studies have reported that OE has various health-promoting functions, such as increased glutathione expression in cells [13], free radical-scavenging activity [14,15], anti-tumor activity [16], and anti-platelet aggregation activity [17]. In addition, our previous reports suggested that OE inhibited the initiator action of carcinogens in mice [18] and accelerated the recovery of proximal tubular epithelial cell function in *p*-aminophenol-induced nephrotoxicity in rats [19]. Moreover, we have previously reported that OE intake alters the composition of gut microbiota and its metabolites including short-chain fatty acids (SCFA) in mice and rats [20,21]. The compositional balance of gut microbiota is closely related to the health of the host [22] and is closely related to IBD [6]. Host–microbe crosstalk plays an important role in maintaining whole body homeostasis, and deterioration of the gut environment such as changes in microbial composition and bacterial metabolism, triggers intestinal inflammation [23,24]. In particular, the compositional alterations of microbiota can affect the availability of substrates to host enzymes, the maturation of host immunity, and the growth of bacteria with virulence characteristics [25]. Thus, changes in the structure and composition of the microbiota in gut are thought to be linked to the initiation and progression of IBD [26]. Dietary OE that alters gut microbiota and SCFA composition [20,21] is expected to have an inhibiting property on the promotion and progression of IBD. However, few studies have focused on the anti-colitis effects of dietary OE on DSS-induced acute experimental colitis in mice. Here, we investigated the anti-colitis effect of OE intake related to gut microbiota and its metabolites in a DSS-induced acute experimental colitis mouse model.

## 2. Materials and Methods

### 2.1. Materials

Fresh raw oysters were heated with hot water (about 90 °C for about 2 h), the solids were removed by filter paper, and the filtrate was dried with a drum dryer (110 °C for 2.25 h). The resulting powder was named OE. The nutritional composition (carbohydrate, glycogen, crude protein, amino acid composition, taurine, crude fat, ash, sodium chloride, and moisture) of OE were measured by Japan Food Research Laboratories (Tokyo, Japan) and are shown in Table 1. DSS was bought from MP Biomedicals (Irvine, CA, USA).

### 2.2. Animal Experimental Approval

The animal experimental protocol was approved (approval no. 1810) after being reviewed by the Animal Ethics Committee of Kansai University.

### 2.3. Animal Experiment

C57BL/6J mice (male, four-week-old) were purchased from Japan SLC Inc. (Hamamatsu, Japan). Mice were kept in a stable air-conditioned room (temperature: 21–23 °C; illuminated, 08:00–20:00). Mice were allowed to have free access to diet and water for the experimental period. After acclimatization for 7 days, the mice were divided into 3 groups so that there were 8 mice in each group (control, control + DSS, and OE + DSS). The control and control + DSS groups were administered the control diet, while the OE + DSS group received the OE diet. Table 2 shows the ingredients of the experimental diets (control and OE) based on American Institute of Nutrition (AIN)-93G composition [27]. The amounts of sodium chloride in the control and OE diets were prepared to be equal. Food intake and body weight (BW) were determined every 2 days for 3 weeks. After 3 weeks, the drinking water in the control + DSS and OE + DSS groups was replaced with 2.5% (*w*/*w*) DSS solution for 7 days. At 10:00 am for 7 days of DSS administration, the disease activity index (DAI) score [28] (stool consistency, relative BW loss, and occult blood in stool) was assessed daily. The time schedule for animal experiment is shown in Figure S1. As >20% BW loss compared to the BW at the start of DSS administration was the humane endpoint in this study; however, no mice fell into this category. One mouse in the control + DSS group died 7 days after DSS administration.

After 7 days of DSS administration, feces from each mouse were collected. Then, the mice (not fasted) were euthanized under isoflurane anesthesia (9:00–11:00 AM). Blood was collected from the inferior vena cava, and serum was obtained by centrifugation (2000× *g*, for 15 min). The liver, kidney, spleen, and colon were quickly removed, weighed, and the colon length was measured. The colonic contents were washed out with cold saline, and the distal colon sections were fixed in 10% formalin solution.

### 2.4. Serum Biochemical Parameters

Aspartate aminotransferase (AST), alanine aminotransferase (ALT), creatine phosphokinase (CPK), lactate dehydrogenase (LDH), total protein, and albumin in serum were measured by Japan Medical Laboratory (Kaizuka, Japan).

### 2.5. Histopathological Analysis

Distal colonic sections, which had been fixed in 10% formalin, were embedded in paraffin and 5-µm sections were prepared. After staining with hematoxylin-eosin, histological analysis (inflammation, extent, regeneration, and crypt damage) was evaluated by a pathologist, as described previously [29].

### 2.6. Fecal SCFA Compositions

Fecal SCFA composition at day 7 after 2.5% DSS administration was analyzed using gas chromatography (GC-2014; Shimadzu Co., Kyoto, Japan) [30].

### 2.7. 16S rRNA Amplicon Sequence and Bioinformatics

For 16S rRNA amplicon sequencing, five fecal samples from the experimental groups were randomly selected and total DNA was extracted using ISOSPIN Fecal DNA (Nippon Gene Co., Ltd., Tokyo, Japan). As described previously report for details [31], 16S rRNA amplicon sequence and raw sequence data analyses were conducted using a next-generation sequencing (NGS) system (Ion PGM^TM^; Thermo Fisher Scientific Inc., Waltham, MA, USA) and Ion Reporter Software (Metagenomics 16S w1.1 ver. 5.14, Thermo Fisher Scientific Inc.) to obtain the relative bacterial composition and diversity. The β-diversity was estimated and visualized using principal component analysis (PCA) using ClustVis (https://biit.cs.ut.ee/clustvis/ (accessed on 12 October 2021)). Linear discriminant analysis effect size (LEfSe) [32] analysis (at levels of LDA scores log_10_ > 4 and *p* < 0.05.) was evaluated using Galaxy (http://huttenhower.sph.harvard.edu/galaxy/ (accessed on 16 October 2021)).

### 2.8. Statistical Analysis

Data are shown as the mean and standard errors of the mean (SEM). For DAI and histopathological scores (ordinal scale), Kruskal-Wallis test followed by uncorrected Dunn’s test were used. For other parameters (ratio scale or interval scale), one-way analysis of variance followed by Holm-Sidak’s multiple comparisons test was used. Group comparisons were analyzed between the control group and the control + DSS group, and between the control + DSS group and the OE + DSS group. The relationships among fecal relative bacteria at the genus level, each SCFA compositions, and the indicators of the severity of DSS-induced colitis were evaluated using Spearman’s correlation coefficient test. *p*-values of less than 0.05 were considered statistically significant, and those of 0.05 ≤ *p* < 0.10 were considered statistically tendency. Statistical analyses were conducted using GraphPad Prism, version 7.0 (GraphPad Software, San Diego, CA, USA).

## 3. Results

### 3.1. OE Intake Alleviates DSS-Induced Acute Experimental Colitis Symptoms

To evaluate the effect of OE on acute experimental colitis symptoms, mice fed an OE diet for 3 weeks were administered DSS in drinking water for 7 days. No significant differences were observed in the food and water intake or DSS solution intake during the experimental period (Table S1). In the acute experimental colitis mice, the relative cecum weights and serum biochemical parameters, including AST, ALT, CPK, LDH, total protein, and albumin were significantly changed (Table S1). On the other hand, compared to the control + DSS group, OE intake had no effect on these parameters.

**Figure 1:**
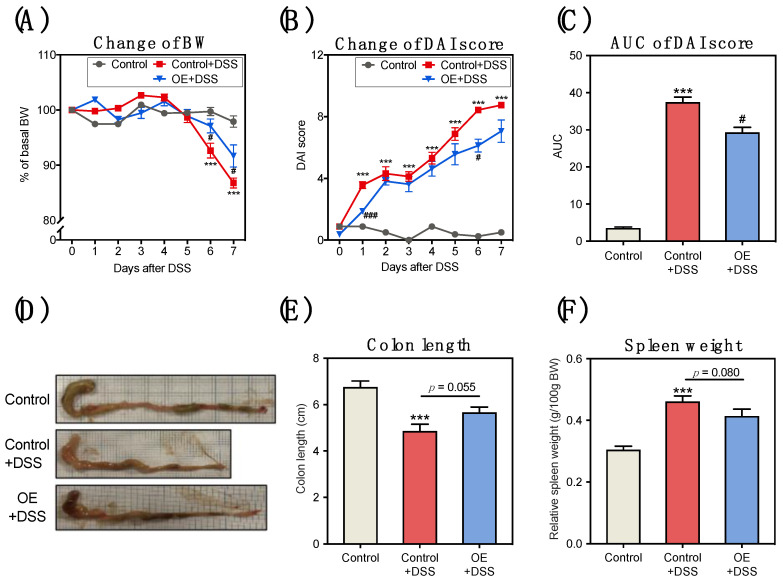
Effect of OE on BW, DAI score, colon length, and relative spleen weight in mice of DSS-induced acute experimental colitis symptoms. (**A**) Changes in BW. Changes in the BW percentage (%) = BW on the specified day/the BW at day 0 × 100. (**B**) Change of DAI score. (**C**) AUC of DAI score of mice during administration of 2.5% DSS in drinking water. (**D**) Representative pictures of colons. (**E**) Colon length of mice at day 7 after 2.5% DSS administration. (**F**) Relative spleen weight of mice at day 7 after 2.5% DSS administration. The values shown are the mean ± SEM (8 mice in control and OE + DSS groups and 7 mice in control + DSS group). *** *p* < 0.001 vs. control group, ^#^ *p* < 0.05, and ^###^ *p* < 0.001 vs. control + DSS group. AUC, area under the curve; BW, body weight; DAI, disease activity index; DSS, dodecyl sodium sulfate; OE, oyster extract; SEM, standard error of the mean.

BW loss and an increased DAI score were observed in the DSS administration group from the start of DSS administration (Figure 1A–C). BW loss in the OE + DSS group was significantly lower on days 6 and 7 compared to the control + DSS group (Figure 1A). The DAI score in the OE + DSS group was significantly lower on days 1 and 6 than in the control + DSS group (Figure 1B). Compared to that in the control + DSS group, the increases in area under the curve (AUC) of the DAI score in the OE + DSS group was attenuated (Figure 1C).

Both shortened colon length and gained relative spleen weight have been used as indirect indicators of the severity of DSS-induced colitis [33]. Compared to the control group, the control + DSS group showed significantly shortened colon length and increased relative spleen weight (Figure 1E,F). Compared with the control + DSS group, the OE intake tended to decrease in the shortened colon length and gained relative spleen weight (*p* = 0.055 and 0.080, respectively). These results indirectly indicated that OE tended to suppress DSS-induced colitis based on changes in BW loss, DAI score, colon length, and relative spleen weight.

### 3.2. OE Intake Alleviates the Histological Damage in the Colon of DSS-Induced Acute Experimental Colitis Mice

**Figure 2:**
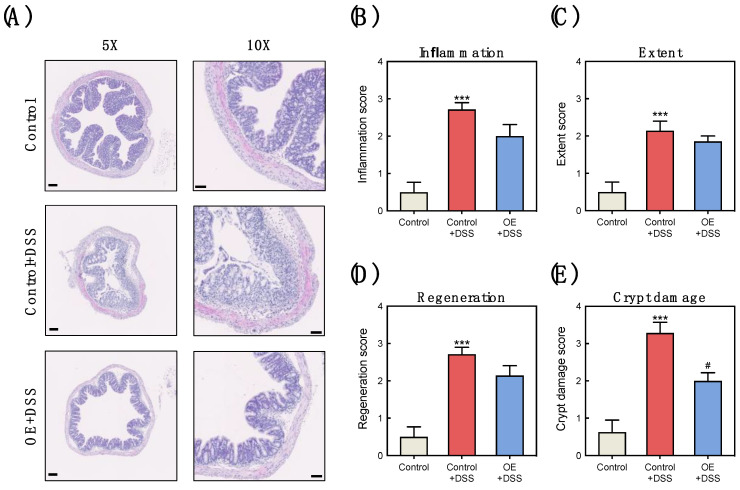
Effect of OE on the histological damages in the colon tissues of DSS-treated mice. (**A**) Representative histologic sections were stained with hematoxylin-eosin. Scale bar, 100 μm (5×) and 50 μm (10×). (**B**–**E**) Histological analysis in the colon of mice at day 7 after 2.5% DSS administration. The values shown are the mean ± SEM (8 mice in control and OE + DSS groups and 7 mice in control + DSS group). *** *p* < 0.001 vs. control group, ^#^ *p* < 0.05 vs. control + DSS group. DSS, dodecyl sodium sulfate; OE, oyster extract; SEM, standard error of the mean.

Figure 2 shows the histological damage of the colon induced by DSS, including inflammation, extent, regeneration, and crypt damage. DSS administration significantly increased histological damage in the colon tissues (Figure 2B–E). Colon crypt damage in the OE + DSS group was significantly attenuated compared with the control + DSS group (Figure 2E).

### 3.3. Effect of OE on SCFA Contents and Compositions in the Fecal of DSS-Induced Acute Experimental Colitis Mice

**Figure 3:**
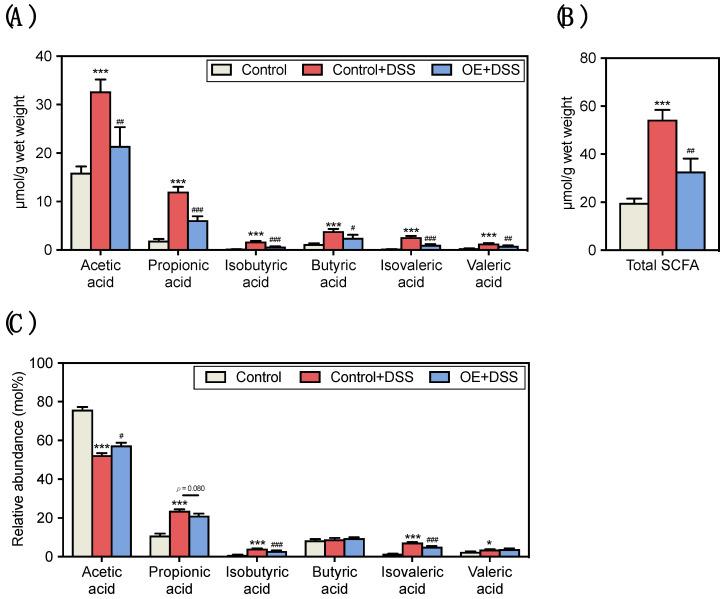
Effect of OE on the SCFA contents and compositions in the feces of DSS-induced acute experimental colitis mice. (**A**) SCFA content, (**B**) total SCFA content, and (**C**) relative SCFA abundance in the feces of mice at day 7 after 2.5% DSS administration. The values shown are the mean ± SEM (*n* = 8 per group). * *p* < 0.05 and *** *p* < 0.001 vs. control group, ^#^ *p* < 0.05, ^##^ *p* < 0.01, and ^###^ *p* < 0.001 vs. control + DSS group. DSS, dodecyl sodium sulfate; OE, oyster extract; SCFA, short-chain fatty acids, SEM, standard error of the mean.

Colonic metabolite profiles have been shown to be linked to the symptoms of IBD [34]. Therefore, we assessed the fecal SCFA content and composition in DSS-treated mice fed an OE diet (Figure 3A–C). The control + DSS group showed higher fecal SCFA and total SCFA content than the control group (Figure 3A,B). In addition, the OE + DSS group showed lower fecal SCFA and total SCFA contents than the control + DSS group. Since the cecal weight was lower in the control + DSS group than the control group (Table S1), which may be indicative of the concentration of SCFA in feces, the relative fecal SCFA composition is also shown in Figure 3C. Compared to the control group, the control + DSS group showed lower relative acetic acid and higher relative propionic acid, isobutyric acid, isovaleric acid, and valeric acid compositions. Moreover, the OE + DSS group showed higher relative acetic acid and lower relative isobutyric acid and isovaleric acid compositions than the control + DSS group. These results suggest that OE can ameliorate the changes in fecal SCFA content and composition induced by DSS administration.

### 3.4. Effect of OE on Microbiota in the Feces of DSS-Induced Acute Experimental Colitis Mice

**Figure 4:**
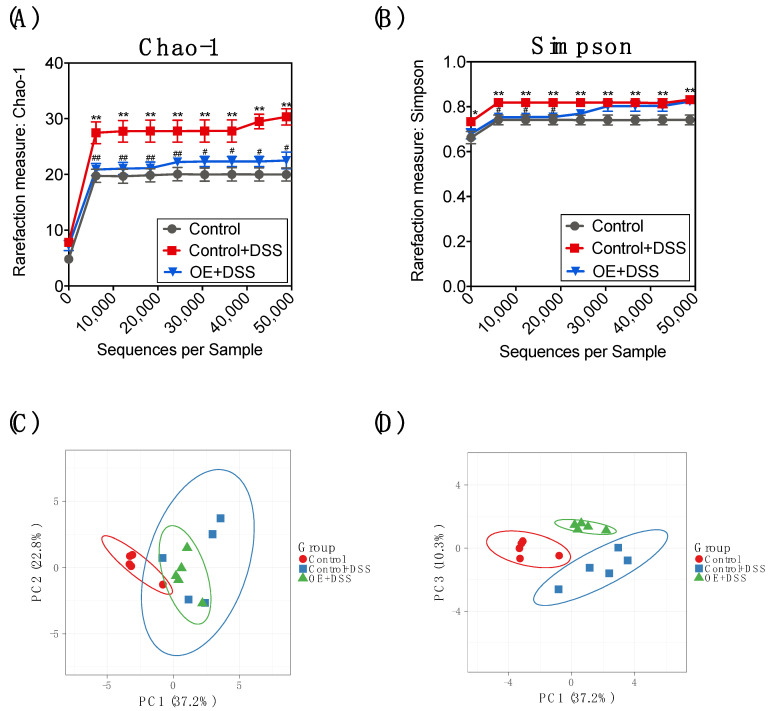
Effect of OE on the α- and β-diversities in the feces of DSS-treated mice. (**A**,**B**) Bacterial rarefaction curves based of the Chao-1 and Simpson induces, respectively. The values shown are the mean ± SEM (*n* = 5 per group). * *p* < 0.05 and ** *p* < 0.01 vs. control group, ^#^ *p* < 0.05 and ^##^ *p* < 0.01 vs. control + DSS group. (**C**,**D**) PCA of the bacterial compositions at the genus level. The prediction ellipse shows the extent to which a new sample falls inside the ellipse with probability 0.95. DSS, dodecyl sodium sulfate; OE, oyster extract, PCA, principal component analysis.

Changes have been reported in the structure and composition of the gut microbiota in the DSS-induced colitis model [6]. To determine whether OE improves the gut microbiota, we approached the structure and composition of microbiota in feces using NGS-based 16S rRNA amplicon sequencing. The number of valid reads after processing by Ion Reporter was not significantly different among the experimental groups (control: 168,632 ± 19,922, control + DSS: 158,437 ± 24,418, and OE + DSS: 127,632 ± 22,393, respectively). Since the rarefaction curves (Chao-1 and Simpson) of each sample are plateaus, the depth of sequencing was sufficient to reflect the diversity, and the result of the sequence were credible (Figure 4A,B). Compared to the control group, the indices of Chao-1 (community richness) and Simpson (community diversity and community evenness) [35] were significantly higher in the control + DSS group. OE intake improved the DSS-induced increase in the Chao-1 index. The β-diversity results (Figure 4C,D) showed that DSS administration changed the structure of fecal microbiota. The OE + DSS group was clearly separated from the control + DSS group at PC1-PC3 at the genus level (Figure 4D).

**Figure 5:**
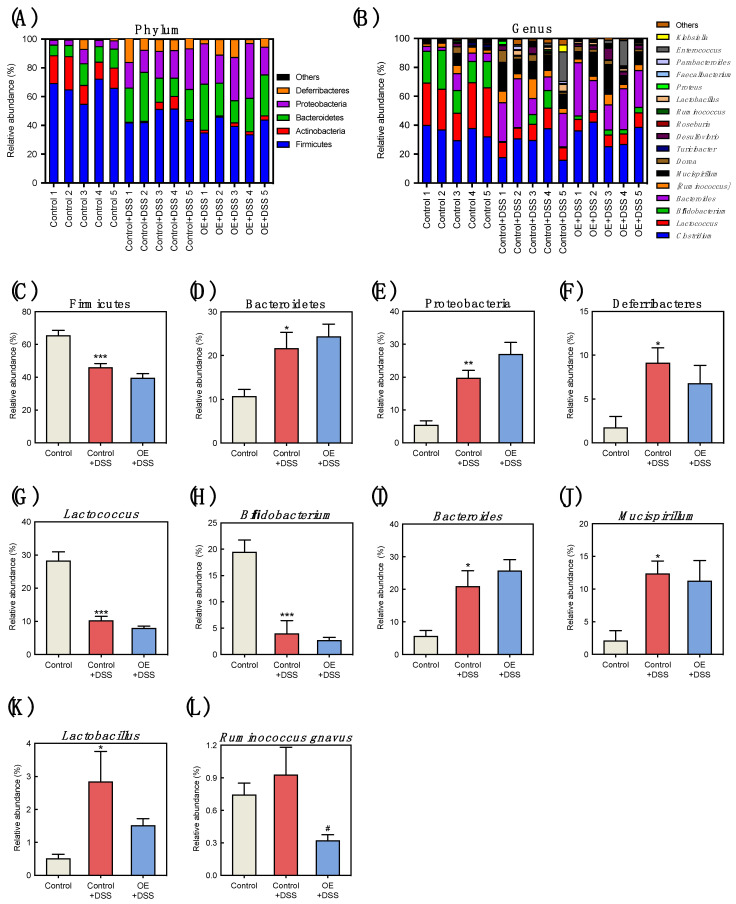
Effect of OE on the microbiota composition in the feces of DSS-induced acute experimental colitis mice. (**A**,**B**) Relative fecal bacteria abundance at the phylum and genus level, respectively. The relative fecal bacteria abundance was sorted from the highest relative abundance in the control group, and those with a relative abundance of less than 0.5% were grouped together as “others”. (**C**–**L**) Relative abundance of each bacteria. The values shown are the mean ± SEM (*n* = 5 per group). * *p* < 0.05, ** *p* < 0.01, and *** *p* < 0.001 vs. control group, ^#^ *p* < 0.05 vs. control + DSS group. DSS, dodecyl sodium sulfate; OE, oyster extract; SEM, standard error of the mean.

Histograms were used to show the relative bacterial abundances at the phylum and genus levels in the feces of the experimental groups (Figure 5A,B). Compared with the control group, DSS administration induced a lower relative Firmicutes abundance and higher relative Bacteroidetes, Proteobacteria, and Deferribacteres abundances (Figure 5C–F). The OE intake did not change relative bacterial abundance at the phylum level compared with the control + DSS group. Compared with the control group, DSS administration induced lower relative *Lactococcus*, and *Bifidobacterium* abundances and higher relative *Bacteroides*, *Mucispirillum*, and *Lactobacillus* abundances at the genus level (Figure 5G–K). Compared to the control + DSS group, the OE + DSS group showed significantly reduced relative abundance of *Ruminococcus gnavus* (Figure 5L).

**Figure 6:**
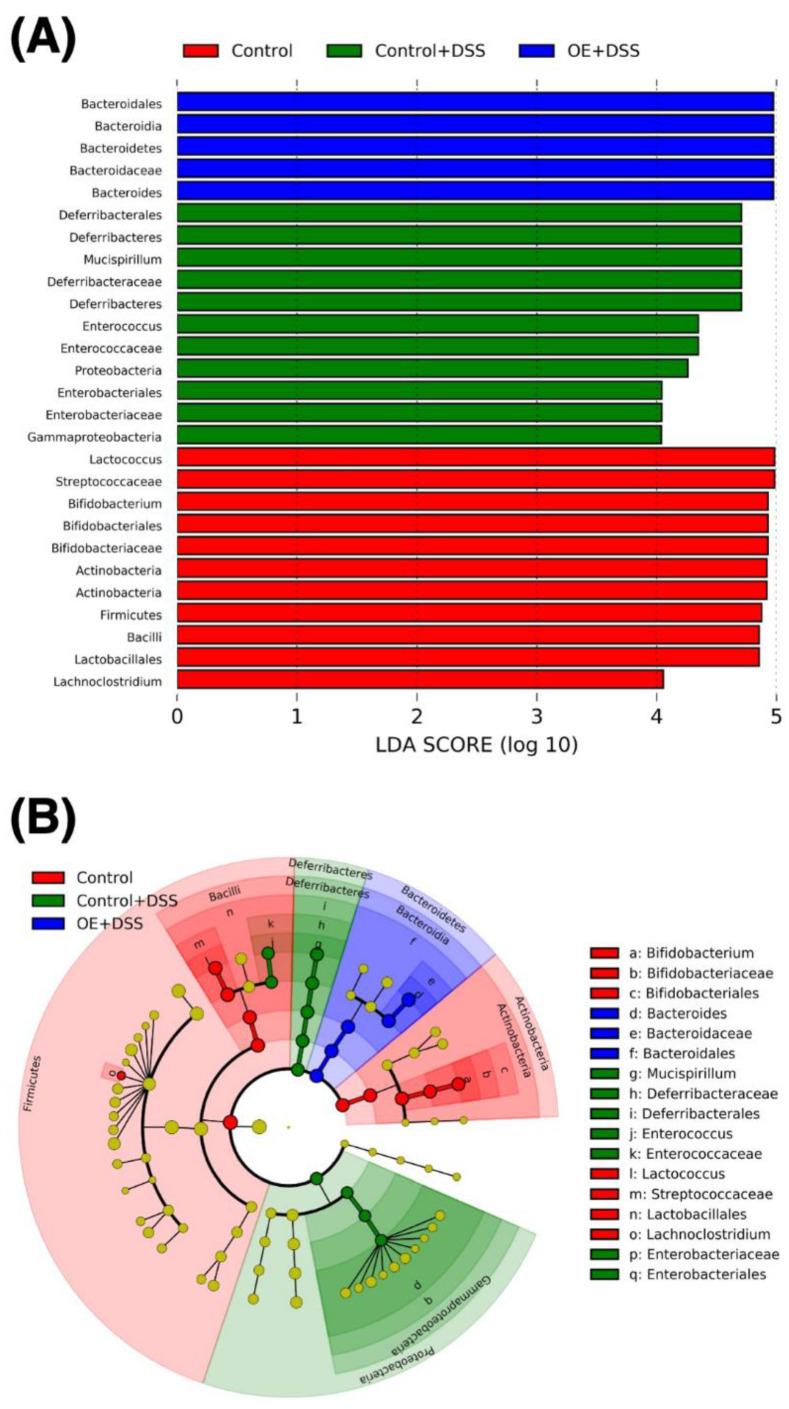
Comparison of microbiota in the feces using LEfSe. The results (**A**) and cladogram (**B**) of the phylogenetic distribution in fecal bacteria among the experimental groups obtained by LefSe analysis are showed. Bacteria are listed at the LDA scores log_10_ > 4 and *p* < 0.05. DSS, dodecyl sodium sulfate; LDA, linear discriminant analysis; LEfSe, linear discriminant analysis effect size; OE, oyster extract.

To examine the effects of OE intake and DSS administration on fecal microbiota, LEfSe analysis was used to identify the bacteria with the greatest differences among the experimental groups (Figure 6). We obtained the 27 dominant microbiota at each level among the experimental groups. The Actinobacteria and Firmicutes phylotypes were found to be more abundant in the control group at the phylum level. In contrast, the Defferibacteres and Proteobacteria phylotypes were more abundant in the control + DSS group. In addition, the Bacteroidetes phylotype was more abundant in the OE + DSS group.

## 4. Discussion

OE has health-promoting properties, including an ability to attenuate nephrotoxicity and the initiator action of the carcinogen, reduce liver cholesterol levels, and alter the gut microbiota composition [18,19,20,21,36]. However, few studies have focused on the effects of dietary OE on DSS-induced acute experimental colitis. In this study, we used a mouse model of DSS-induced acute experimental colitis to simulate clinical colitis. After a 7-day induction with 2.5% DSS administration, the watery stool, reduced BW, shortened colon length, and gained relative spleen weight in the mice of the DSS administrated groups indicated that the acute experimental colitis model was successfully established. Since serum albumin level, an indicator of nutritional status, has been reported to be reduced in patients with IBD [37], it is thought that serum total protein, AST, and LDH levels decreased due to worsening nutritional status. In addition, the high serum CPK levels observed in the control + DSS group can be due to the muscle damage induced by DSS [38]. Other researchers have also reported that it is possible to induce a mouse model of acute experimental colitis models using methods similar to those reported in this study [39,40]. A series of studies have shown that food components have favorable ameliorative effects on acute colitis [8,9,10,11,12]. We conducted the first study on the attenuation of acute experimental colitis by OE. Compared to the control + DSS group, BW loss, increased DAI score, colonic shortening (*p* = 0.055), increased relative spleen weight (*p* = 0.080), and increased histological score (crypt damage) were found to be attenuated in the OE + DSS group, indicating that dietary OE inhibited acute experimental colitis induced by DSS administration (Figure 1 and Figure 2).

SCFA in the gut work as the energy source for colonic epithelial cells and are metabolites released by intestinal bacteria that utilize non-digestible carbohydrates and proteins [41]. SCFA are closely linked to gut inflammation [42]. For example, butyric acid plays a role in the protection and improvement of intestinal barrier function [43]. IBD patients have decreased numbers of SCFA-producing bacteria and SCFA content in their feces [44], and the production of SCFA is linked to a reduced IBD risk [45]. It has been reported that SCFA content in the colon and feces is decreased in mice with colitis [45], and DSS administration was found to increase the fecal total SCFA content in the present study (Figure 3B). SCFA absorption and metabolism have been reported to be impaired in patients with inflammatory colonic mucosa [46] and active ulcerative colitis [47]. In addition, acetic acid has been used to induce colitis in murine models [48]. In the present study, strong correlations were observed between the fecal SCFA content and indicators of the severity of DSS-induced colitis (Figure S2). Therefore, the increased fecal total SCFA and acetic acid contents observed with DSS administration may be linked to colonic progression and dysfunction of acute experimental colitis. On the other hand, OE intake decreased the total fecal SCFA content compared to the control + DSS group (Figure 3B). This decrease in the fecal SCFA content by OE intake indicates a reduction in the severity of colitis, which may be due to the absorption of SCFA in the colon by reducing crypt damage (Figure 2E).

Branched SCFA (mainly isobutyric acid and isovaleric acid) account for about 5–10% of the total SCFA content in gut [49]. It has been reported that the reduction of SCFA production with prebiotic intake, isovaleric acid, and isobutyric acid induces apoptosis, and isobutyric acid may be involved in sodium absorption by affecting ion exchange; however, its role is not completely understood [49]. Colonic branched SCFA were increased in lipopolysaccharide-induced colitis mice [50], and isovaleric acid was increased in advanced colorectal cancer in humans [51]. In addition, isobutyric acid and isovaleric acid, which are mainly produced from the branched-chain amino acids fermentation, are considered to be markers of protein fermentation [52]. The branched SCFA in the feces increased by DSS administration were thought to be substrates of unabsorbed branched-chain amino acids derived from casein in the control and OE diets (200 and 182.3 g/kg, respectively). In this study, compared with the control + DSS group, OE intake decreased fecal isobutyric acid and isovaleric acid contents and compositions (Figure 3A,C). This phenomenon could be partly due to the fact that OE intake alleviated colitis and aggravated protein digestion by DSS administration.

A notable reduction in the α-diversity of the gut microbiota was shown in the DSS-treated mice [53]. However, the control + DSS group showed a higher α-diversity, including the Chao-1 and Simpson indices, compared to the control group (Figure 4A,B). This phenomenon may be because DSS administration decreased the number of dominant bacteria (Firmicutes) and increased the number of other bacteria (Bacteroidetes, Proteobacteria, and Deferribacteres) under these experimental conditions. The β-diversity results at PC1-PC3 (Figure 4D) clearly showed the separation of the OE + DSS group from the control + DSS group. Although OE intake could not reverse the effect on the microbiota in the feces by DSS administration, OE intake seemed to regulate the fecal microbiota in DSS-treated mice. These findings suggest that OE intake, compared with the control diet in DSS-induced colitis mice, had different effects on the fecal microbiota structure at the genus level.

Fecal relative acetic acid and propionic acid compositions were strongly correlated with the indicators of the severity of DSS-induced colitis (Figure S3) and relative *Bifidobacterium*, *Mucispirillum*, and *Lactobacillus* abundances (*p* < 0.01; Figure S4). Not only the amount of fecal SCFA, but also their compositional ratio related to the severity of colitis. These SCFA (acetic acid and propionic acid) are produced by the intestinal bacteria [54]. *Bifidobacterium* species produce large amounts of acetic acid during sugar fermentation [55], while *Lactobacillus* produced the highest amount of propionic acid and the least amount of acetic acid [56]. The change in the relative acetic acid and propionic acid compositions could be related to the abundance of *Bifidobacterium* and *Lactobacillus*.

OE intake was found to decrease the proportion of harmful bacteria, such as *R. gnavus*. *R. gnavus* forms colonies on the surface of the intestinal mucosa, where it can utilize the sialic acid of mucin sugar chains as a carbon source [57]. A large-scale microbiome sequencing study showed that the IBD patients have a high relative *R. gnavus* abundance compared to healthy individuals [58]. *R. gnavus* abundance usually makes up less than 0.1% of the gut microbiota in the healthy intestine; however, *R. gnavus* abundance may rise transiently as the disease flares up in some patients with IBD [59]. A relative abundance of *R. gnavus* was observed in the feces of DSS-treated mice fed OE compared with those treated only with DSS administration (Figure 5L), suggesting that OE intake could protect against acute colitis due to its regulatory effect on harmful bacteria, such as *R. gnavus*. However, this hypothesis will need to be verified in more in-depth studies. At present, it is difficult to assess the exact proportion of bacteria at the species level using short-read 16S rRNA amplicon sequencing.

The OE is composed of a variety of components. Therefore, it is difficult to identify the specific compound in OE that causes the attenuation of acute experimental colitis induced by DSS administration. The first possible component that has protective effects against acute colitis is glycogen, which is found in 32.5 g/100 g of OE. It has been reported that the intake of glycogen (enzymatically synthesized) markedly increased SCFA production in the cecum, which may be due to assimilation by microbiota, especially *Bifidobacterium* and *Lactobacillus* [60]. The next possible component that has protective effects against acute colitis is taurine in OE (OE contained 5.5 g/100 g taurine). Taurine has been shown to have a preventive effect on experimental colitis induced by DSS administration [61], regulating the immune response and restoring the intestinal tight junction barrier [11]. The identification of components in OE that have preventive effects against acute colitis will require further studies on the effects of glycogen and taurine in OE in DSS-induced colitis.

## 5. Conclusions

OE exerted protective effects against DSS-induced acute experimental colitis, in part through the alteration of fecal SCFA content and composition and modulation of the gut microbiota. These results contribute to our understanding of the protective effect of OE in alleviating acute experimental colitis and provide insights for its use in the development and use of functional food ingredients.

## Figures and Tables

**Table 1:** Composition of the oyster extract.

	Oyster Extract
	**g/100 g**
Carbohydrate	50.6
Glycogen	34.5
Crude protein	28.1
Amino acid composition	
Alanine	1.10
Arginine	0.52
Aspartic acid ^1^	1.09
Cystine	0.14
Glutamic acid ^2^	2.35
Glycine	1.24
Histidine	0.25
Isoleucine	0.25
Leucine	0.40
Lysine	0.51
Methionine	0.17
Phenylalanine	0.23
Proline	1.22
Serine	0.41
Threonine	0.45
Tryptophan	0.06
Tyrosine	0.19
Valine	0.33
Taurine	5.5
Crude fat	2.0
Ash	15.4
Sodium chloride	8.2
Moisture	3.9

^1^ Aspartic acid + Asparagine. ^2^ Glutamic acid + Glutamine.

**Table 2:** Composition of the experimental diets.

	Experimental Diets ^1^	
	Control	OE
	g/100 g	
Dextrinized corn starch	132	132
Corn starch	397.486	366.486
Sucrose	95.9	100
Cellulose	50	50
Casein	200	182.3
L-Cystine	3	3
Choline bitartrate	2.5	2.5
Sodium chloride-free AIN-93G mineral mixture	35	35
AIN-93 vitamin mixture	10	10
Soybean oil	70	68.7
Oyster extract powder	-	50
Sodium chloride	4.1	-
*tert*-Butylhydroquinone	0.014	0.014

^1^ Based on the AIN-93G composition, diets were prepared. The amount of sodium chloride in the control and OE diets was equal. AIN, American Institute of Nutrition; OE, oyster extract.

## Data Availability

The data presented in this study are available on request from the corresponding author, upon reasonable request.

## Supplementary Materials

The following are available online at https://www.mdpi.com/article/10.3390/foods11030373/s1, Figure S1: Schematic diagram of DSS-treated mice, Figure S2: Heatmap representation of Spearman’s correlation coefficient between fecal each SCFA contents and the indicators of the severity of DSS-induced colitis, Figure S3: Heatmap representation of Spearman’s correlation coefficient between fecal environments (relative bacteria and each SCFA compositions) and the indicators of the severity of DSS-induced colitis, Figure S4: Heatmap representation of Spearman’s correlation coefficient between relative bacteria and each SCFA compositions in the feces of DSS-treated mice; Table S1: Growth parameters, organ weights, and serum biochemical parameters in DSS-treated mice.
