# Supplementary material for: Oyster (Crassostrea gigas) Extract Attenuates Dextran Sulfate Sodium-Induced Acute Experimental Colitis by Improving Gut Microbiota and Short-Chain Fatty Acids Compositions in Mice
Source: Foods. 2022 Jan 27;11(3):373. doi: 10.3390/foods11030373 (PMC8834099; doi:10.3390/foods11030373)
Supplement: Supplementary file 1 [file foods-11-00373-s001.zip › foods-1519367-supplementary.pdf]

Supplementary Materials

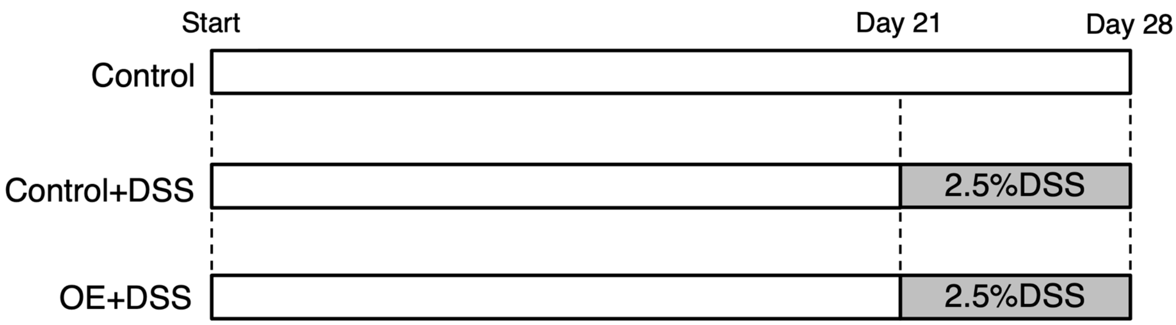

Figure S1. Schematic diagram of DSS-treated mice. DSS, dodecyl sodium sulfate; OE, oyster extract.

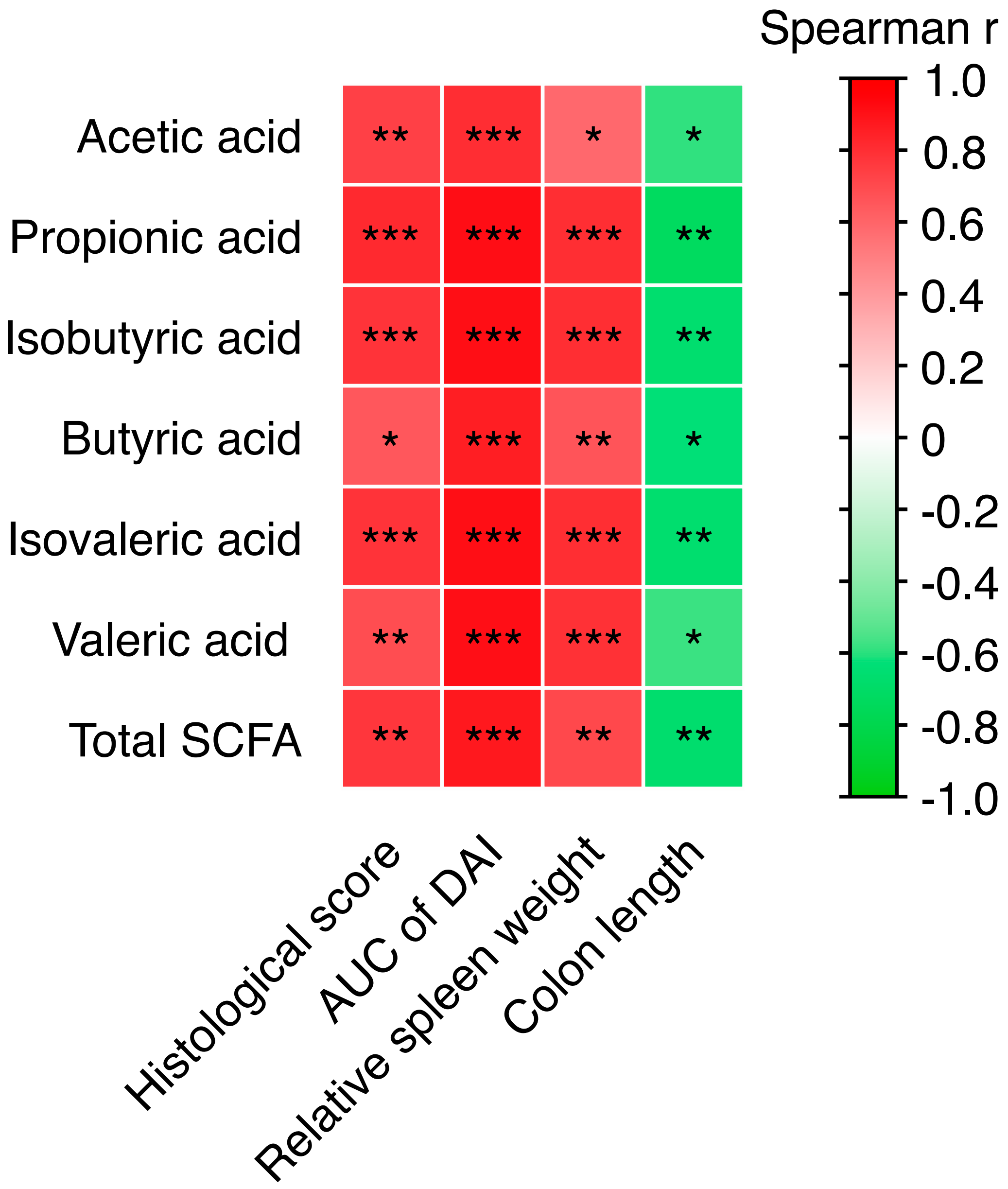

Figure S2. Heatmap representation of Spearman's correlation coefficient between fecal each SCFA contents and the indicators of the severity of DSS-induced colitis. Red: position correlations; Green: negative correlations. * $p < 0.05$, ** $p < 0.01$, and *** $p < 0.001$.

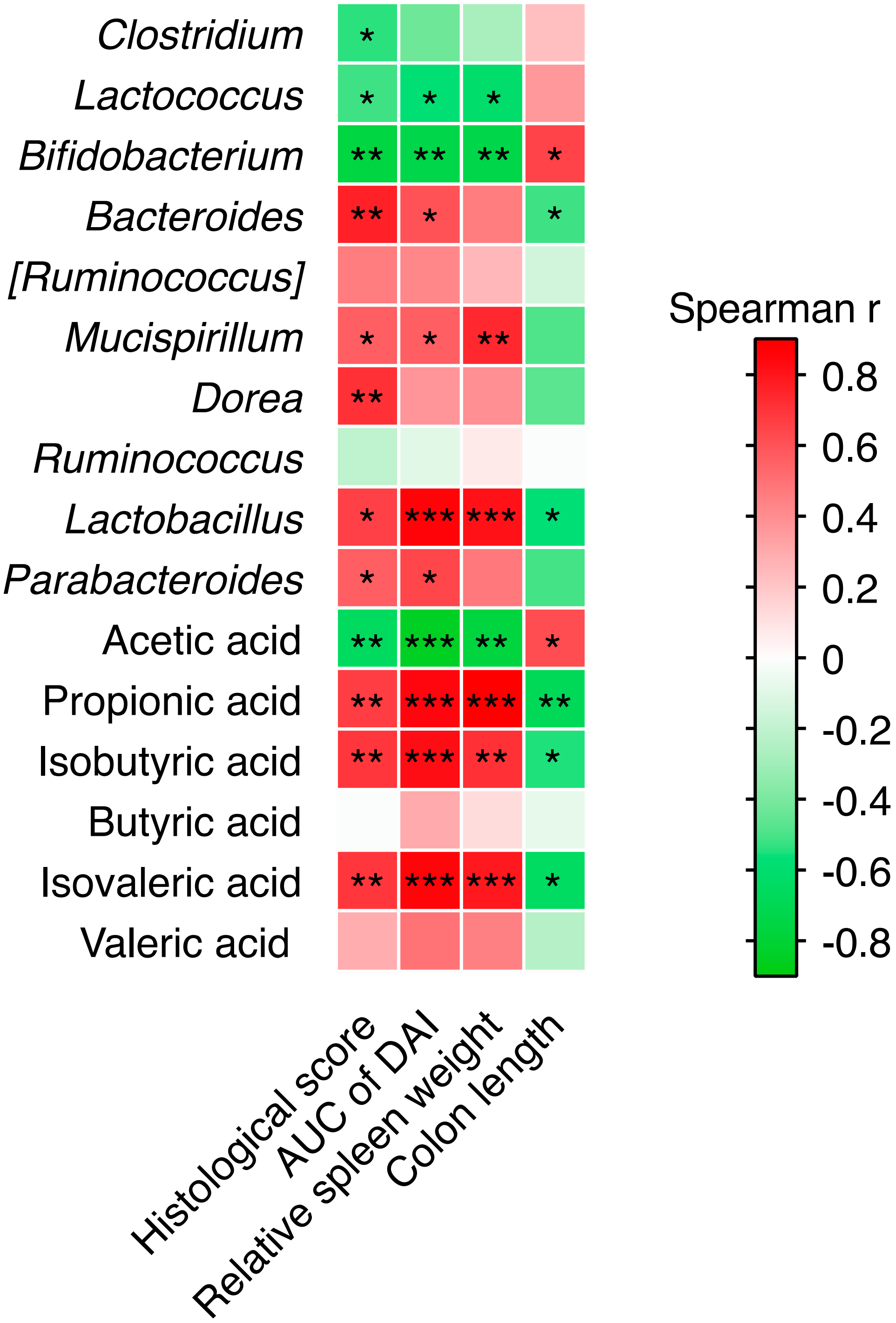

Figure S3. Heatmap representation of Spearman's correlation coefficient between fecal environments (relative bacteria and each SCFA compositions) and the indicators of the severity of DSS-induced colitis. Red: position correlations; Green: negative correlations. * $p < 0.05$, ** $p < 0.01$, and *** $p < 0.001$.

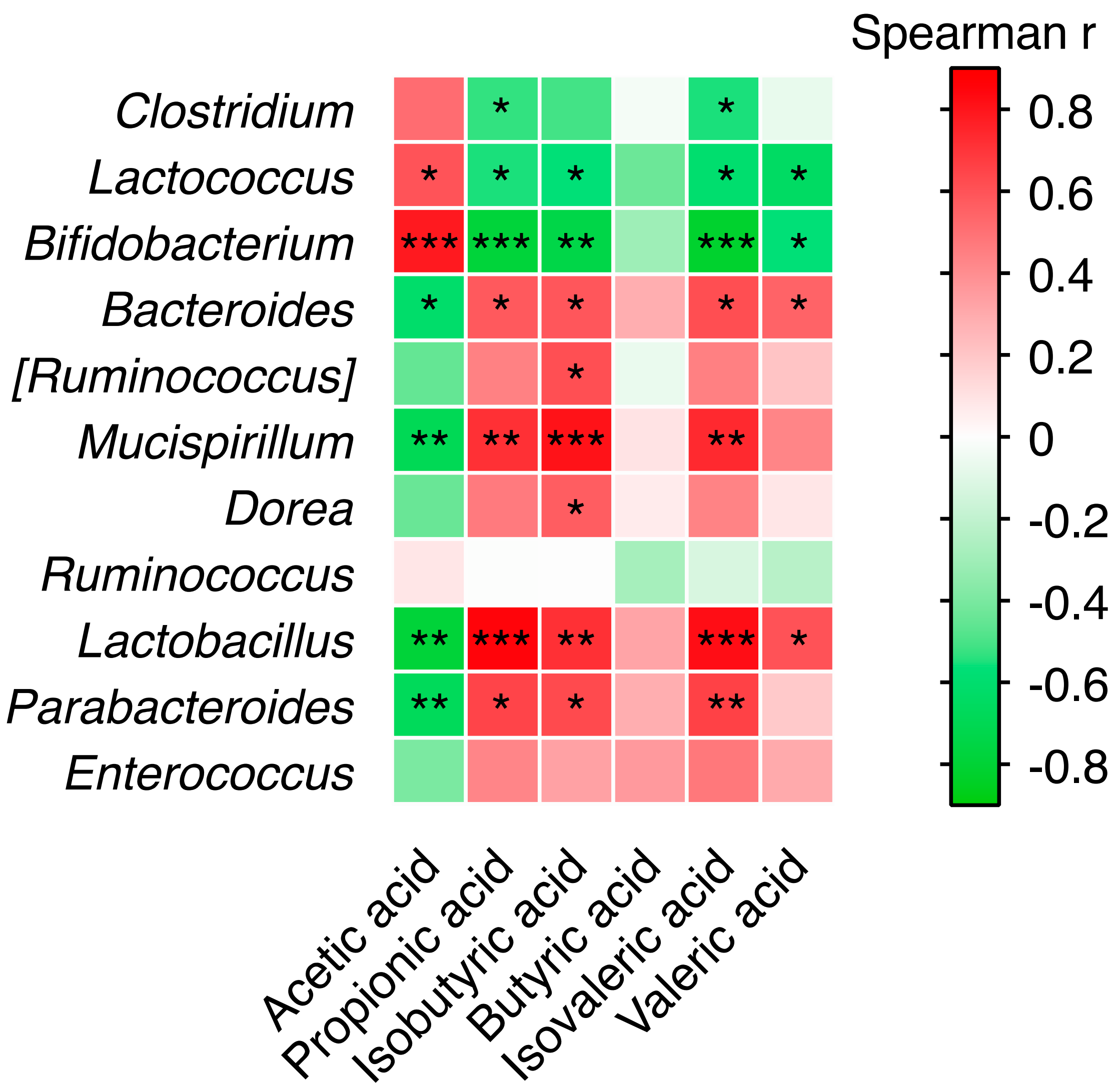

Figure S4. Heatmap representation of Spearman's correlation coefficient between relative bacteria and each SCFA compositions in the feces of DSS-treated mice. Red: position correlations; Green: negative correlations. * $p < 0.05$, ** $p < 0.01$, and *** $p < 0.001$.

Table S1. Growth parameters, organs weights, and serum biochemical parameters in DSS-treated mice.

	Groups		
	Control	Control+DSS	OE+DSS
Growth parameters			
Food intake (g/day)	2.56 ± 0.08	2.40 ± 0.17	2.37 ± 0.13
Water intake (g/day)	8.24 ± 0.74	7.85 ± 0.67	7.47 ± 0.65
DSS solution intake (g/day)	-	9.58 ± 0.50	9.06 ± 0.52
Organ weights (g/100g BW)			
Liver	4.19 ± 0.17	4.52 ± 0.16	4.40 ± 0.18
Kidney	1.16 ± 0.03	1.19 ± 0.02	1.16 ± 0.01
Cecum	0.97 ± 0.11	0.57 ± 0.05 **	0.80 ± 0.11
Serum biochemical parameters			
Total protein	4.63 ± 0.05	3.79 ± 0.10 ***	3.85 ± 0.13
Albumin	2.81 ± 0.04	2.06 ± 0.07 ***	1.96 ± 0.07
AST	42.6 ± 1.6	34.9 ± 1.2 **	40.8 ± 2.8
ALT	15.0 ± 1.5	11.1 ± 1.0	11.4 ± 1.8
CPK	33.9 ± 1.5	53.1 ± 4.7 **	45.5 ± 7.2
LDH	176 ± 17	147 ± 21	150 ± 17

The values shown are the mean ± SEM (n = 7-8 per group). Data were analyzed using one-way analysis of variance followed by Holm-Sidak's multiple comparisons test between the control vs. control+DSS and control+DSS vs. OE+DSS groups. ** $p < 0.01$ and *** $p < 0.001$ vs control group. ALT, alanine aminotransferase; AST, aspartate aminotransferase; BW, body weight; CPK, creatine phosphokinase; DSS, dodecyl sodium sulfate; LDH, lactate dehydrogenase.
